# Enhancing levan biosynthesis by destroying the strongly acidic environment caused by membrane-bound glucose dehydrogenase (mGDH) in *Gluconobacter* sp. MP2116

**DOI:** 10.1016/j.synbio.2024.08.005

**Published:** 2024-08-20

**Authors:** Junjie Tian, Shumin Wei, Wenxing Liang, Guangyuan Wang

**Affiliations:** a College of Life Sciences, Shandong Province Key Laboratory of Applied Mycology, Qingdao Agricultural University, Changcheng Road, No.700, Qingdao, 266109, China; b College of Plant Health and Medicine, The Key Laboratory of Integrated Crop Pest Management of Shandong Province, Qingdao Agricultural University, Qingdao, 266109, China

**Keywords:** Levan, Levansucrase, Acidification, Membrane-bound glucose dehydrogenase

## Abstract

Levan produced by *Gluconobacter* spp. has great potential in biotechnological applications. However, *Gluconobacter* spp. can synthesize organic acids during fermentation, resulting in environmental acidification. Few studies have focused on the effects of environmental acidification on levan synthesis. This study revealed that the organic acids, mainly gluconic acid (GA) and 2-keto-gluconic acid (2KGA) secreted by *Gluconobacter* sp. MP2116 created a highly acidic environment (pH < 3) that inhibited levan biosynthesis. The levansucrase derived from strain MP2116 had high enzyme activity at pH 4.0 ∼ pH 6.5. When the ambient pH was less than 3, the enzyme activity decreased by 67 %. Knocking out the *mgdh* gene of membrane-bound glucose dehydrogenase (mGDH) in the GA and 2KGA synthesis pathway in strain MP2116 eliminated the inhibitory effect of high acid levels on levansucrase function. As a result, the levan yield increased from 7.4 g/l (wild-type) to 18.8 g/l (Δ*mgdh*) during fermentation without pH control. This study provides a new strategy for improving levan production by preventing the inhibition of polysaccharide synthesis by environmental acidification.

## Graphical abstract

**Figure:**
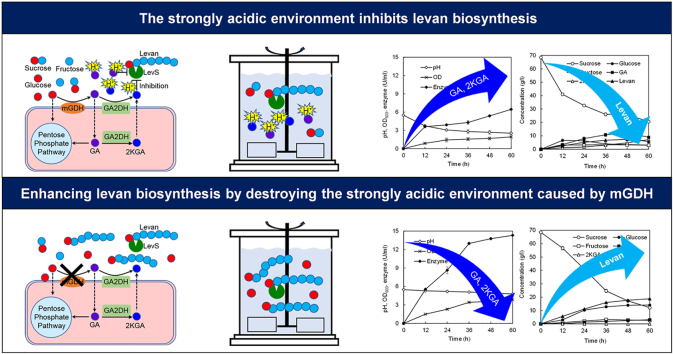

## 1 Introduction

Levan, a well-known natural beta-2,6-fructan, is considered a promising multipurpose material in the biomedical, food, modern cosmetology, and nanotechnology industries due to its wide range of distinct properties, including antibacterial, antiviral, and prebiotic activities, stabilization effects, film-forming capacity, specific viscosity, and high compatibility [[1], [2], [3]]. Levan biosynthesis is catalyzed by bacterial extracellular levansucrase (EC 2.4.1.10) [4], which belongs to the glycoside hydrolase 68 (GH 68) family [1,5]. In general, sucrose is used as the substrate for levan synthesis [6]. Using the free energy from cleavage of the substrate sucrose [7,8], levansucrase catalyzes microbial levan biosynthesis by transferring the fructosyl unit from sucrose to the polyfructose chain while continuously releasing glucose [4,9]. When water is used as an acceptor instead of the growing polyfructose, levansucrase catalyzes the hydrolysis of sucrose [10,11]. The released glucose can be used for microbial cell metabolism. Since bacterial levansucrase catalyzes fructan biosynthesis in the extracellular environment [4,12], the external environment (e.g., pH) can affect levan biosynthesis.

To date, various bacterial species of genera such as *Gluconobacter*, *Bacillus*, *Erwinia*, *Lactobacillus, Zymomonas*, *Pseudomonas*, and *Azotobacter* have been intensively used to produce levan [2,13,14]. Among these microorganisms, the genus *Gluconobacter* has proven to be a reliable platform for levan production because of its generally recognized as safe (GRAS) status, ease of genetic modification, and adaptability [2,12]. Previous reports have investigated the strong levan formation ability of several *Gluconobacter* strains, such as *Gluconobacter albidus* TMW 2.1191 [4,15,16], *Gluconobacter japonicus* LMG 1417 [12], *Gluconobacter nephelii* P1464 [17], *Gluconobacter* sp. TMW 2.767 and *Gluconobacter cerinus* DSM 9533T [18]. *Gluconobacter* spp. are capable of synthesizing a variety of organic acids and secreting them into the environment, causing environmental acidification. However, the relationship between environmental acidification and polysaccharide synthesis is unclear.

*Gluconobacter* strains are characterized by the incomplete oxidation of various sugars and alcohols by membrane-bound dehydrogenases [[19], [20], [21]]. The membrane-bound glucose dehydrogenase (mGDH) of *Gluconobacter* is responsible for the oxidation of glucose [22,23]. Due to the presence of a large amount of glucose in levan synthesis [24], the glucose released from sucrose is easily oxidized by mGDH to gluconic acid (GA). The resulting GA is further oxidized to 2-keto-gluconic acid (2KGA) and 5-keto-gluconic acid (5KGA) [25,26], resulting in a decrease in the extracellular pH. Therefore, it is highly important to investigate the relationship between the extracellular pH and levan biosynthesis.

In the present study, the effect of extracellular acidification on polysaccharide synthesis in *Gluconobacter* sp. MP2116 was investigated. Disruption of the *mgdh* gene reduced the carbon flux of organic acid synthesis, which was accompanied by the avoidance of the inhibition of polysaccharide synthesis by the acidic extracellular environment and the enhancement of levan production in strain MP2116.

## 2 Materials and methods

### 2.1 Strains, plasmids, and media

The *Gluconobacter* strains used in this study were MP2116 and Δ*mgdh*. Strain MP2116, the wild-type [24], was maintained on potato extract dextrose agar (PDA) slants at 4 °C. The strain Δ*mgdh*, an *mgdh* gene deletion mutant of MP2116, was grown on PDA slants supplemented with 0.01 % kanamycin. The *Escherichia coli* DH5α strain used for plasmid recovery and the *E. coli* BL21(DE3) strain used for the production of the recombinant levansucrase were grown in Luria–Bertani (LB) broth. The pMD19-T vector purchased from TaKaRa (DaLian) was used for DNA sequencing. The pET-28a vector was used for heterologous gene expression. The seed culture was grown in PDB medium, which contained all the ingredients of the PDA medium except agar. The medium used for levan production (LP medium) contained 68.5 g/l sucrose, 0.9 g/l mannitol, 5 g/l yeast extract, and 3 g/l casein peptone, as previously described with appropriate modifications [12].

### 2.2 Evaluation of the ability of *Gluconobacter* sp. MP2116 to accumulate exopolysaccharide (EPS)

Strain MP2116 was first inoculated into 50 ml of LP medium supplemented with different carbon sources (68.5 g/l), such as sucrose, glucose, fructose and mannitol. After inoculation with 5 mL of MP2116 seed culture, the cells were incubated in a shaker at 180 rpm and 28 °C for 48 h. After removing the cells by centrifugation at 12,000×*g* for 10 min, the EPS in the supernatant was precipitated by adding 95 % (v/v) ethanol (supernatant:ethanol = 1:4 [v/v]) [12]. Thereafter, the EPS was recovered by centrifugation and dried [27]. Then, the weight of the EPS was measured. Cell growth was measured according to the optical density of the culture broth at 600 nm [26]. The levansucrase activity in the supernatant of the fermentation broth was determined as described below.

### 2.3 Heterologous expression and purification of the recombinant levansucrase

For enzyme characterization, levansucrase from strain MP2116 was heterologously expressed in *E. coli* BL21(DE3). The *levs* gene from strain MP2116 with a His_6_-tag was first amplified by PCR using the primers 28LevS-F/R (Table S1), which were designed according to previously described sequences (Fig. S1) [24]. After digestion with *Nde* I, the resulting fragments were ligated into the plasmid pET-28a using a One Step Cloning Kit (Vazyme Biotech), yielding pET-28a-*levs*. The expression vector pET-28a-*levs* was finally introduced into *E. coli* BL21(DE3) cells by heat shock transformation for heterologous expression.

For the production of recombinant levansucrase, BL21(DE3) cells containing pET-28a-*levs* were first grown in LB broth (1000 ml) supplemented with 0.01 % kanamycin at 37 °C and 160 rpm until the cell density (OD_600_) reached 0.6. After adding IPTG (at a final concentration of 0.1 mM) for induction, the culture was incubated at 160 rpm and 16 °C for 20 h. The cells were harvested by centrifugation at 12,000×*g* and 4 °C for 5 min. The obtained cells were then resuspended in 10 ml of lysis buffer followed by sonication on ice. After centrifugation at 12,000×*g* and 4 °C for 15 min, the His_6_-tagged recombinant levansucrase in the supernatant was purified by nickel affinity chromatography. Then, the purity of the purified levansucrase was analyzed by SDS‒PAGE. The concentration of the purified levansucrase was determined according to a Bradford protein assay kit (Sangon Biotech).

### 2.4 Determination of levansucrase activity

Sucrose, dissolved in 50 mM sodium acetate buffer (pH 5.0) at a concentration of 200 g/l, was used as the substrate for the enzyme reaction. The reaction system contained 0.1 ml of enzyme and 0.9 ml of sucrose solution. After incubation at 30 °C for 15 min, the reaction was terminated by boiling in water for 10 min. The amount of glucose produced by the reaction mixture was measured using a glucose assay kit (Sangon Biotech) [24]. One levansucrase unit (U) was defined as the amount of enzyme that releases 1 μmol of glucose from sucrose in 1 min [28,29].

### 2.5 Assay of the purified levansucrase reaction conditions

The impact of pH on the purified enzyme activity was determined by performing the reaction at different pH values by using citrate buffer (pH 2.0–3.0), acetate buffer (pH 4.0–5.0) and phosphate buffer (pH 6.0–8.0). To address the impact of temperature, various incubation temperatures (from 10 °C to 65 °C) were used in for the reaction. The activity of levansucrase was then determined.

### 2.6 Construction of the *mgdh* gene deletion construct

To knock out the *mgdh* gene in strain MP2116, the *mgdh* gene was first cloned from genomic DNA of strain MP2116 using the primers mGDH-F/R (Table S1) which were designed according to the *mgdh* sequences of *G. japonicus* LMG 1417 (accession no. NZ_LHZJ01000078.1/WP_010501939.1) and *Gluconobacter frateurii* ML.ISBL3 (accession no. CP092689.1/UMM08771.1). Then, two flanking fragments of *mgdh* were amplified by PCR from the genomic DNA of strain MP2116 with the primer pairs Up-F/R and Down-F/R (Table S1). The kanamycin resistance gene (*kana*) containing promoter was derived by PCR using the primers Kana-F/R from the plasmid pNA1312-GY [30]. Thereafter, the two flanking sequences and the *kana* gene were fused by splice overlap PCR with the primers Up-F/Down-R (Table S1). The resulting deletion cassette (2.5 kb, *up::kana::down*) was ligated into the pMD19-T vector for sequencing. Finally, the obtained deletion cassette was transformed into strain MP2116 via electroporation [31]. Positive transformants were selected on PDA plates supplemented with 0.01 % kanamycin. The gene deletion was confirmed by PCR amplification using the primers in-F/R, Kana-F/R, and out-F/R (Table S1).

### 2.7 Levan production in a 5-L fermentor

Strains MP2116 and Δ*mgdh* were cultured for levan production. For preparation of the seed cultures, the strains MP2116 and Δ*mgdh* were first inoculated into PDB medium and cultured at 180 rpm and 28 °C for 24 h. Then, 200 ml of each seed culture was transferred to 3600 ml of LP medium in a 5-l jar fermentor (Baoxing, China). All the cultures were incubated at 28 °C and aerated at 1.0 volume of medium per minute (VVM) with an agitation speed of 180 rpm. The pH of the culture was not controlled or was maintained at 5.5 by automatic titration with 1 M NaOH. A subset of samples was taken at regular temporal intervals to analyze cell growth, EPS, levansucrase activity, sucrose, glucose, fructose, GA, 2KGA, and 5KGA. Cell growth, EPS and levansucrase activity were determined as described above. The residual sucrose concentration was estimated as described previously [24]. The contents of monomeric sugars and organic acids in the culture were assayed by HPLC as described below.

### 2.8 Determination of the organic acid levels

The organic acids secreted by strains MP2116 and Δ*mgdh* were measured by HPLC (Waters/e2695, USA). In brief, after removing the cells in the culture by centrifugation at 12,000×*g* for 10 min, the supernatants were separated on an Aminex HPX-87H column (Bio-Rad, 300 × 7.8 mm). The sample volume was 5 μl. The elution buffer was 5 mM H_2_SO_4_. The elution speed was set at 0.5 ml/min. The organic acids were detected by an ultraviolet detector at 210 nm.

### 2.9 Monosaccharide composition analysis of levan

The absolute acid hydrolysis of the obtained levan was first performed according to previously described methods [32]. Briefly, the extracted EPS (0.2 g) was hydrolyzed by incubating in 2 ml of 0.1 M HCl at 100 °C for 1 h. The released monomeric sugars were analyzed using HPLC (Waters/e2695, USA) with a Supersil NH2–S column (5 μm, 4.6 × 250 mm) (Dalian Elite Analytical Instruments Co., Ltd.). The sample volume was 10 μl. The elution was acetonitrile and H_2_O (80:20, v/v) with a flow rate of 0.8 ml/min. The released sugars were detected using a refractive index detector. The level of polymerized levan (within a 1 % error) was calculated to be equal to 0.9 times the total monomeric sugars released from the precipitated EPS [32].

### 2.10 Structural characterization of levan

The structure of the obtained EPS was characterized by both ^13^C nuclear magnetic resonance (NMR) (Bruker, Avance III HD 500) and fourier transform infrared (FT-IR) spectroscopy (Thermo Fisher, Nicolet iS10) according to previously described procedures [24,33].

## 3 Results

### 3.1 Sucrose was required the synthesis of EPS by *Gluconobacter* sp. MP2116

**Fig. 1:**
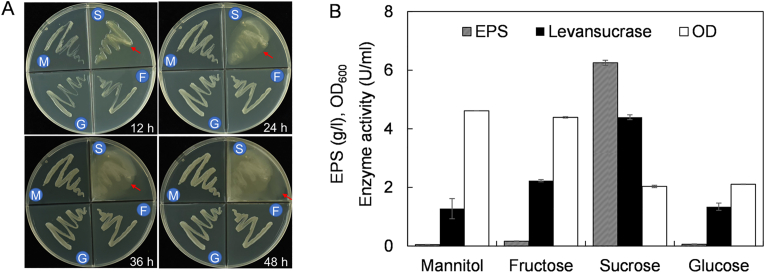
Effect of different carbon sources on EPS production. **(A)** The colonies of MP2116 on plates containing glucose/G, fructose/F, mannitol/M or sucrose/S. The EPS layer is indicated by red arrows. **(B)** Impacts of carbon sources on EPS accumulation, enzyme activity and cell growth. The data are presented as the means ± SDs; n = 3.

To test the EPS production capacity of strain MP2116, we inoculated the strain on plates containing glucose, fructose, mannitol or sucrose. As shown in Fig. 1A, strain MP2116 could form large amounts of EPS around the colonies on sucrose plates but did not form the polysaccharide layer on glucose, fructose or mannitol plates. Consistent with these results, sucrose was the most suitable for levansucrase accumulation and EPS production (Fig. 1B). Strain MP2116 could produce 6.3 g/l EPS and accumulate 4.6 U/ml extracellular levansucrase (Fig. 1B) after growing in LP medium supplemented with sucrose in flask for 48 h. In addition, mannitol and fructose were more beneficial to cell growth than were sucrose and glucose (Fig. 1B). Levan synthesis requires sucrose as a substrate, which is one of the characteristics of fructan synthesis. These results indicated that *Gluconobacter* sp. MP2116 is a strong EPS producer, and the EPS produced might be a levan-type fructan.

### 3.2 Monosaccharide composition and structural characterization of the obtained EPS

**Fig. 2:**
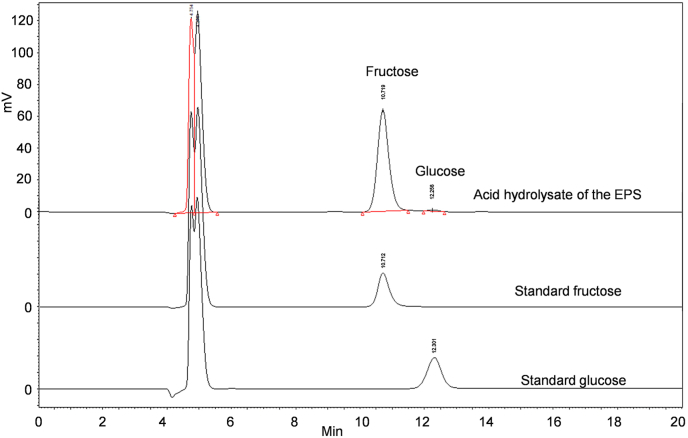
Profiles of monosaccharide composition.

HPLC analysis of the acid hydrolysis of the obtained levan confirmed the major monosaccharide in the sample was fructose (98.9 %) (Fig. 2). In addition, a small amount of glucose (1.1 %) was also detected in the sample (Fig. 2). After calculation, more than 85.8 % of the polymerized fructose was found in the precipitated EPS.

**Fig. 3:**
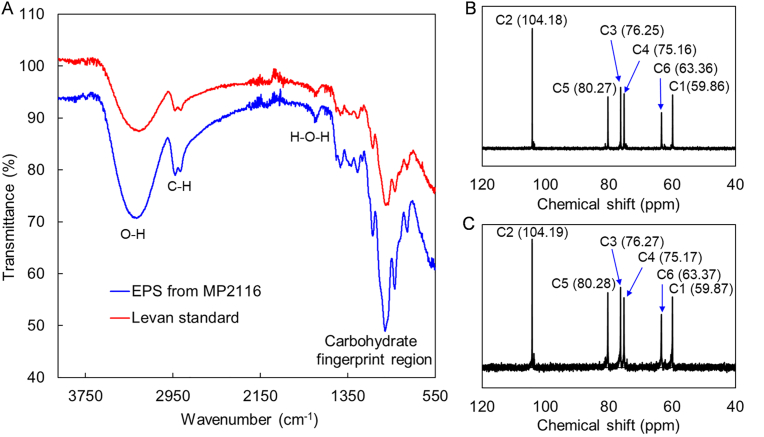
Structural analysis of polysaccharides. **(A)** FT-IR analysis of the EPS produced by strain MP2116. **(B)**^13^C NMR spectrum of the EPS from strain MP2116. **(C)**^13^C NMR spectrum of the standard levan from *E. herbicola*.

Structural analysis of the obtained EPS was further performed by FT-IR and ^13^C NMR. As shown in Fig. 3A, the characteristic absorption bands at 3260 cm^−1^ (O–H stretching), 2929 cm^−1^ (C–H stretching) and 1647 cm^−1^ (H–*O*–H stretching) were found in the EPS from strain MP2116, which exhibited approximately the same characteristic peaks as those of standard levan. The ^13^C NMR spectrum of the EPS produced by *Gluconobacter* sp. MP2116 showed six distinct resonances at 104.18 (C2), 80.27 (C5), 76.25 (C3), 75.16 (C4), 63.36 (C6) and 59.86 (C1) ppm (Fig. 3B), which were characteristic of the levan from *Erwinia herbicola* (Fig. 3C). Taken together, these data confirmed that the EPS produced by *Gluconobacter* sp. MP2116 was a levan-type fructan.

### 3.3 Both extremely acidic (pH < 3) and alkaline (pH > 7) environments inhibited levansucrase activity

**Fig. 4:**
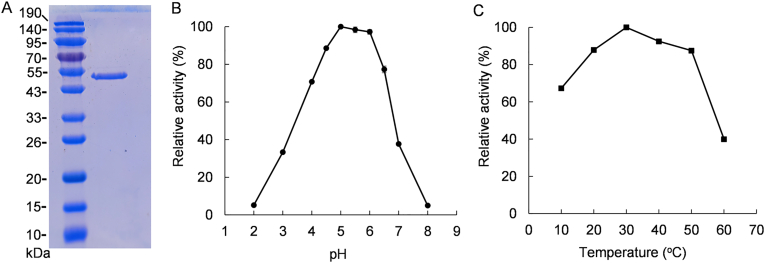
Properties of levansucrase. The data in (B) and (C) are shown as the mean ± SDs; n = 6. **(A)** SDS‒PAGE. **(B)** Optimal reaction pH. **(C)** Optimal reaction temperature.

To produce levan-type fructan from strain MP2116, it is necessary to investigate the enzymatic properties of levansucrase. The levansucrase gene (*levs*) of strain MP2116 was first heterologously expressed in *E. coli* BL21(DE3). After purification, the recombinant enzyme had a molecular weight of approximately 51 kDa (Fig. 4A), which was consistent with our expectation. As shown in Fig. 4B, the purified levansucrase had high activity from pH 4.0 to pH 6.5, and the optimal activity was observed at pH 5.0. At ambient pH values of 5.5 and 6.0, the recombinant levansucrase showed relative activities of 98 % and 97 %, respectively (Fig. 4B). However, when the ambient pH was less than 3 or greater than 7, the activity of the recombinant enzyme significantly decreased (Fig. 4B). Furthermore, the recombinant enzyme had high activity in the temperature range of 20 °C–50 °C (Fig. 4C).

### 3.4 Inactivation of mGDH reduced organic acid production

**Fig. 5:**
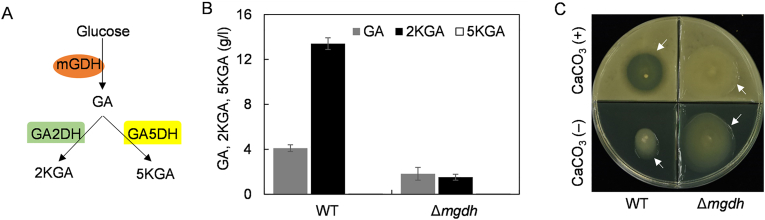
Effects of *mgdh* deletion on organic acid production in flasks. **(A)** Proposed metabolic pathway for GA, 2KGA and 5KGA synthesis in strain MP2116. **(B)** Comparison of the organic acid levels of strains MP2116 and Δ*mgdh* in flasks. The data are presented as the means ± SDs; n = 3. **(C)** Formation of the transparent zone caused by strain MP2116 on plates supplemented with 0.5 % calcium carbonate. The EPS layer is indicated by white arrows.

*Gluconobacter* spp. produce levan in the presence of sucrose but also acidify the culture broth by glucose oxidation producing GA and its derivatives 2KGA and 5KGA. The mGDH is a key enzyme in the GA, 2KGA and 5KGA biosynthesis pathway (Fig. 5A). We speculated that the deletion of *mgdh* gene could reduce the carbon flux of organic acid biosynthesis, increase the extracellular pH, and thus increase the level of levan synthesis. To test this hypothesis, the *mgdh* gene was cloned and subsequently disrupted in *Gluconobacter* sp. MP2116 using the procedures as described above. It was found that the *mgdh* gene of strain MP2116 contained 2421 bp, which showed 98.64 % and 89.76 % identities with its homologs of *G. japonicus* LMG 1417 and *G. frateurii* ML.ISBL3, respectively (Fig. S2). The *mgdh* gene of strain MP2116 was then subsequently knocked out, and PCR amplification showed that one mutant Δ*mgdh* showed physical evidence of *mgdh* deletion (Fig. S3).

To investigate the effect of *mgdh* gene deletion on organic acid production, batch fermentations of the mutant Δ*mgdh* and the wild-type strain MP2116 were performed in flasks using LP medium. After 48 h of incubation, the organic acids in the fermented broth were mainly GA and 2KGA, and almost no 5KGA accumulation was detected in the fermented media (Fig. 5B). Disruption of the *mgdh* gene reduced GA production from 4.1 g/l to 1.8 g/l, and 2KGA production from 13.4 g/l to 1.6 g/l (Fig. 5B). We further inoculated the wild-type strain MP2116 and the mutant Δ*mgdh* on plates containing calcium carbonate. After 24 h of incubation, the calcium carbonate around the colony of the wild-type strain MP2116 was dissolved, while the mutant Δ*mgdh* had no such a zone (Fig. 5C), confirming that destruction of the *mgdh* gene reduced the organic acid yield. More importantly, Δ*mgdh* was able to form a larger EPS layer than MP2116 (Fig. 5C), indicating that the EPS yield significantly increased with decreasing organic acid content.

### 3.5 Effect of acidification on levan biosynthesis

**Fig. 6:**
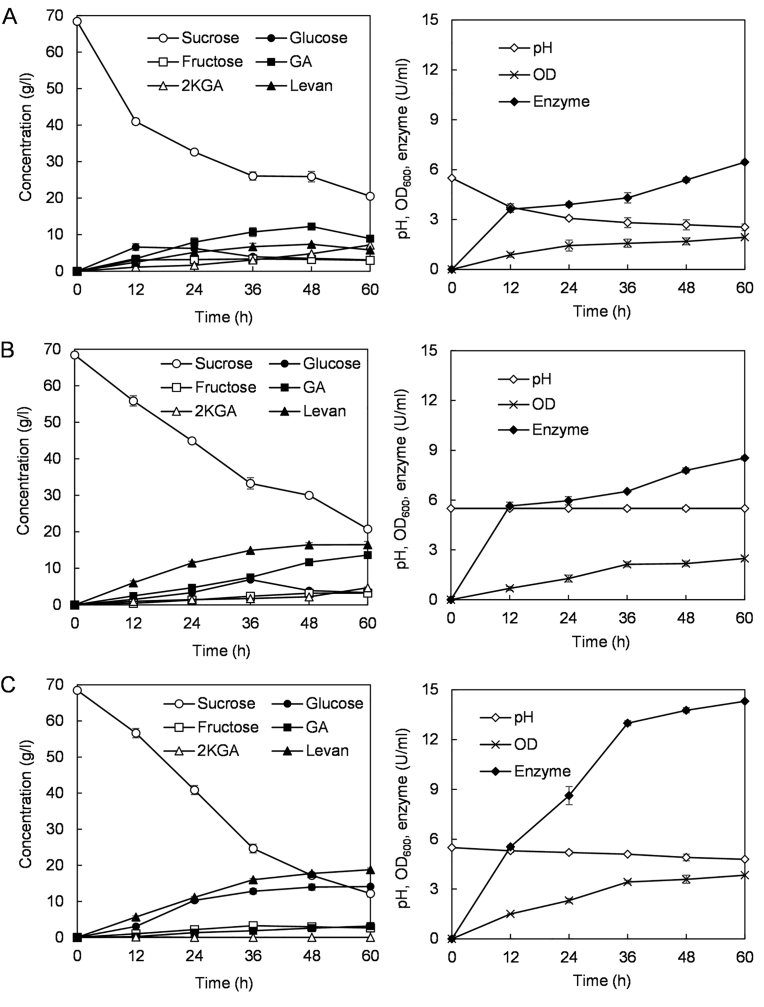
Levan production by the strains MP2116 and Δ*mgdh* in the 5-L fermentor. The data are shown as the means ± SDs (n = 3). **(A)** The fermentation pH of strain MP2116 was not controlled. **(B)** The extracellular pH of strain MP2116 was maintained at 5.5. **(C)** The fermentation profiles of strain Δ*mgdh*.

Since pH had a great influence on levansucrase activity (Fig. 4B), the relationship between the acidic environment caused by *Gluconobacter* sp. MP2116 and levan production was studied. Fermentation was first carried out without adjusting the pH. As shown in Fig. 6A, within 24 h of fermentation, the pH decreased from 5.5 to 3.6, after which the fermentation broth was maintained in a strongly acidic environment (about pH 2.5). Consistent with the results of the shake flask (Fig. 5B), GA and 2KGA were the main components causing acidification of the fermented broth, and 5KGA was hardly detected. When the extracellular pH was not controlled, 7.4 g/l levan, 12.2 g/l GA and 4.7 g/l 2KGA in the culture of strain MP2116 were obtained within 48 h (Fig. 6A). After 60 h of fermentation, the extracellular levansucrase activity reached 6.4 U/ml (without fermentation pH control, Fig. 6A). In contrast, when the culture pH was stabilized at 5.5, the enzyme activity and levan yield significantly increased (Fig. 6B). As seen in Fig. 6B, 7.7 U/ml levansucrase activity and 16.5 g/l levan were obtained from the culture of strain MP2116 after 48 h of fermentation.

The acid production capacity was significantly reduced in the *mgdh* gene deletion mutant (Fig. 5B), the extracellular pH and levan production by Δ*mgdh* were therefore investigated. As shown in Fig. 6C, during the 60-h fermentation process of Δ*mgdh*, the extracellular pH was always maintained above 4.8. In the fermented broth of Δ*mgdh*, a small amount of GA (less than 3.2 g/l) was detected, and 2KGA was almost undetectable (Fig. 6C). Due to the lack of *mghd*, the glucose content in fermentation broth of Δ*mgdh* was significantly higher than that of the wild-type strain (Fig. 6). A slightly acidic environment facilitates the activity of levansucrase (Fig. 4B). The extracellular enzyme activity of Δ*mgdh* reached 14.3 U/ml within 60 h (Fig. 6C). Accordingly, the yield of levan increased significantly, reaching a maximum of 18.8 g/l (Fig. 6C).

Taken together, these findings indicated that the highly acidic extracellular environment produced by strain MP2116 was unfavorable for levan biosynthesis, while the deletion of *mghd* gene was beneficial for reducing the production of organic acids, thereby increasing the yield of levan.

## 4 Discussion

Due to the potential applications of levan in multiple fields, the production of levan by microorganisms has been extensively studied. Previous studies have revealed that *Gluconobacter* spp. are good candidates for levan production [12,16,17,34]. The comparison of various *Gluconobacter* strains for their levan yields was shown in Table 1. As shown in Table 1, the strains used in this study were also potential candidates for levan production.

**Table 1:** Comparison of various *Gluconobacter* strains for levan production.

Strain	Sucrose (g/l)	Levan (g/l)	Yield (g/g)	Process	Reference
MP2116	68.5	16.5	0.24	Fermentor	This study
Δ*mgdh*	68.5	18.8	0.27	Fermentor	This study
*G*. *albidus* TMW 2.1191	80	18.11	0.23	Bioreactor	[16]
*G. japonicus* LMG 1417	607.6	157.9	0.26	Cell-free production	[12]
*G. frateurii* TMW 2.767	80	11.9	0.15	Flask	[34]
*G. cerinus* DSM 9533	80	6.3	0.08	Flask	[34]
*G. nephelii* P1464	135	26.78	0.20	Bioreactor	[17]

In general, the synthesis of levan by bacteria occurs in the extracellular space [4]. Levan synthesis is therefore affected by extracellular conditions such as temperature and pH. The pH during fermentation by *Gluconobacter* strains usually decreases due to organic acid formation. Thus, levan production by *Gluconobacter* spp. could be influenced by changes in pH during the fermentation process. However, there are few studies in which the polysaccharide yield has been increased by blocking organic acid synthesis by genetic engineering. In this study, a key gene in organic acid synthesis, *mgdh*, was knocked out. Acid production by the gene deletion mutant was significantly reduced, which prevented the acidic environment from having adverse effects on levan synthesis, thus greatly increasing polysaccharide production.

Levansucrase activity is affected by environmental pH. Generally, the levansucrase activity is optimal under slightly acidic conditions (pH range 4.5–7.0), while strongly acidic conditions are obviously unfavorable to enzyme activity, as indicated by many studies [5,35]. A levansucrase from *G. japonicus* LMG 1417 showed optimal activity at pH 5, and its activity decreased by 40 % at pH 3 [12]. The optimum pH of the levansucrases from *Bacillus amyloliquefaciens* and *Bacillus licheniformis* was found to be pH 6 [29,36,37]. After the levansucrase of *B. amyloliquefaciens* BH072 was heterologously expressed in *Bacillus subtilis* 168, the recombinant enzyme had only 5.1–5.7 % relative enzyme activity at pH values of 3 and 4 [38]. Consistent with these observations, the optimal reaction condition for levansucrases from strain MP2116 was also a weakly acidic environment (pH 5.0), and a strongly acidic environment (pH < 3.0) greatly inhibited enzyme activity (Fig. 4B).

The most direct way to avoid the adverse effect of a strongly acidic environment on levan production is to control the fermentation pH. In bioreactors, extracellular pH can be maintained by adding acid or alkali. By controlling the fermentation process pH, the levan yield was increased from 7.4 g/l (without pH control) to 16.5 g/l (with pH control at 5.5) (Fig. 6A and B). This strategy has been applied to the production of levan in multiple microbial strains, such as *G. albidus* TMW 2.1191 [16], *Halomonas smyrnensis* AAD6^T^ [39], and *B. subtilis* M [40]. Nevertheless, it is more promising to use genetic engineering to reduce the acid production level of *Gluconobacter* strains.

In the process of levan synthesis, a large amount of byproduct glucose is produced [24]. This glucose can be oxidized to GA by the mGDH of *Gluconobacter* strains, which is subsequently converted to 2KGA and 5KGA [22,25,26]. *Gluconobacter* is a genus of acetic acid bacteria (AAB) [41]. Due to the ability of *Gluconobacter* species to oxidize a wide variety of substances such as sugars, sugar alcohols, and sugar acids, they are an outstanding platform for biotechnological processes [42]. In addition to the organic acids mentioned above, *Gluconobacter* strains can also produce 2-keto-l-gulonic acid [43,44], 3,4-dihydroxybutyric acid [45], 3-hydroxypropionic acid [46], acrylic acid [47], arabinonic acid [48], 1,3-dihydroxyacetone [49], and so on. In this study, *Gluconobacter* sp. MP2116 mainly produced GA and 2KGA. Knocking out the *mgdh* gene in strain MP2116 significantly reduced the levels of organic acid. As a result, the polysaccharide synthesis ability of the mutant Δ*mgdh* was greatly improved (Fig. 6C).

In addition, the mutant Δ*mgdh* grew faster than the wild-type strain MP2116 (Fig. 6). Consistent with these results, knocking out *ga5dh* and *ga2dh* also increased the growth rate of *G. japonicus* CGMCC 1.49 [26]. These genes, *mgdh*, *ga5dh* and *ga2dh*, are all located in the same metabolic pathway (Fig. 5A). However, it is still unknown why disrupting the *mgdh* gene would promote the cell growth in this study.

**Fig. 7:**
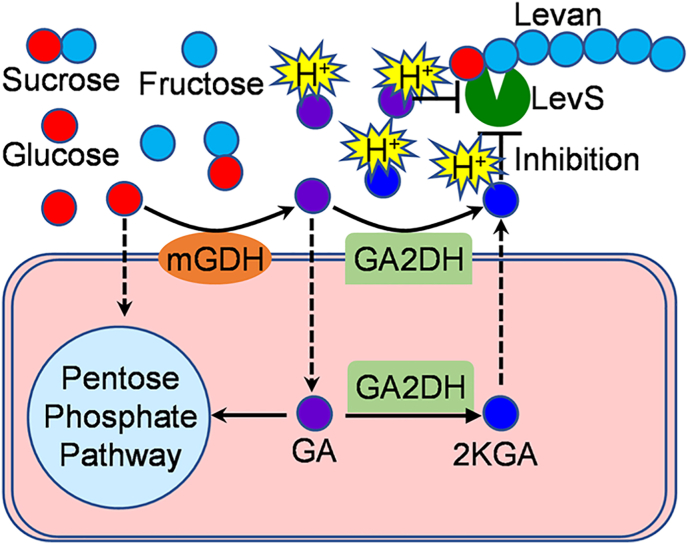
Schematic overview of levan synthesis in response to acidic environment in *Gluconobacter* sp. MP2116.

There are two types of gluconate-2-dehydrogenase (GA2DH) in *Gluconobacter*, one in the periplasm and the other inside the cell [26]. Based on the above discussion, we constructed a diagram of the relationship between acidic environments and polysaccharide synthesis in strain MP2116 (Fig. 7). As shown in Fig. 7, strain MP2116 caused the formation of a high-acid environment, which inhibited levan synthesis.

## 5 Conclusions

In this study, *Gluconobacter* sp. MP2116 was found to be a potential candidate for levan production. However, the process of levan synthesis was inhibited by the acidic environment produced by strain MP2116. One of the key genes in the pathway of GA and 2KGA synthesis, *mgdh*, was knocked out, which decreased the production of GA and 2KGA. In the 5-L fermentations (without pH control), the levan yields of strains MP2116 and Δ*mgdh* reached 7.4 g/l and 18.8 g/l, respectively. Our findings indicated that the use of an engineered strain with weakened acid production could address the challenges posed by an acidic extracellular environment for levan synthesis in strain MP2116, thereby increasing the levan yield.

## CRediT authorship contribution statement

**Junjie Tian:** Writing – original draft, Validation, Methodology, Investigation, Formal analysis, Data curation, Conceptualization. **Shumin Wei:** Visualization, Validation, Software, Methodology, Investigation, Formal analysis. **Wenxing Liang:** Writing – review & editing, Funding acquisition. **Guangyuan Wang:** Writing – review & editing, Writing – original draft, Supervision, Resources, Investigation, Funding acquisition, Conceptualization.

## Declaration of competing interest

The authors declare that they have no known competing financial interests or personal relationships that could have appeared to influence the work reported in this paper.
