# Supplementary material for: Enhancing levan biosynthesis by destroying the strongly acidic environment caused by membrane-bound glucose dehydrogenase (mGDH) in Gluconobacter sp. MP2116
Source: Synth Syst Biotechnol. 2024 Aug 20;10(1):68–75. doi: 10.1016/j.synbio.2024.08.005 (PMC11388042; doi:10.1016/j.synbio.2024.08.005)
Supplement: Multimedia component 1 [file mmc1.doc]

**Table S1** The primers used in this study.

| Primer | Sequence (5'-3') |  |
| --- | --- | --- |
| 28LevS-F | CTGGTGCCGCGCGGCAGCCATATGAATGCTATTTCCAGCCGAATTC |  |
| 28LevS-R | CAGTGGTGGTGGTGGTGGTGCTCAGGCGCGAACGTCATAGGCCATAGA |  |
| mGDH-F | ATGAGCACATCYTCCCGGCCAGGGC |  |
| mGDH-R | TCAGTTRCCCAGYGARTAGGCAATC |  |
| Up-F | TCCAGAGCAATACGGTAGTTC |  |
| Up-R | GTGCTTGCGGCAGCGTGAAGCTAGCTGATGTTCCTAGAGCTGTTAT |  |
| Down-F | TCGCCTTCTTGACGAGTTCTTCTGAGACGGCTTTACAGGATTCATC |  |
| Down-R | AGGCGAGGGTTGTTGTTTTGAGAA |  |
| Kana-F | GCTAGCTTCACGCTGCCGCAAGCAC |  |
| Kana-R | TCAGAAGAACTCGTCAAGAAGGCGA |  |
| in-F | CCGCTCCGATATCGTAATCATTCTC |  |
| in-R | ACCGACAGGCCACCCCATTCGAACA |  |
| out-F | TGCTTGATGGTCTTAGCGT |  |
| out-R | ATCGCTGCCACTGTGTGAT |  |
