# Supplementary figures and images for: Enhancing levan biosynthesis by destroying the strongly acidic environment caused by membrane-bound glucose dehydrogenase (mGDH) in Gluconobacter sp. MP2116
Source: Synth Syst Biotechnol. 2024 Aug 20;10(1):68–75. doi: 10.1016/j.synbio.2024.08.005 (PMC11388042; doi:10.1016/j.synbio.2024.08.005)

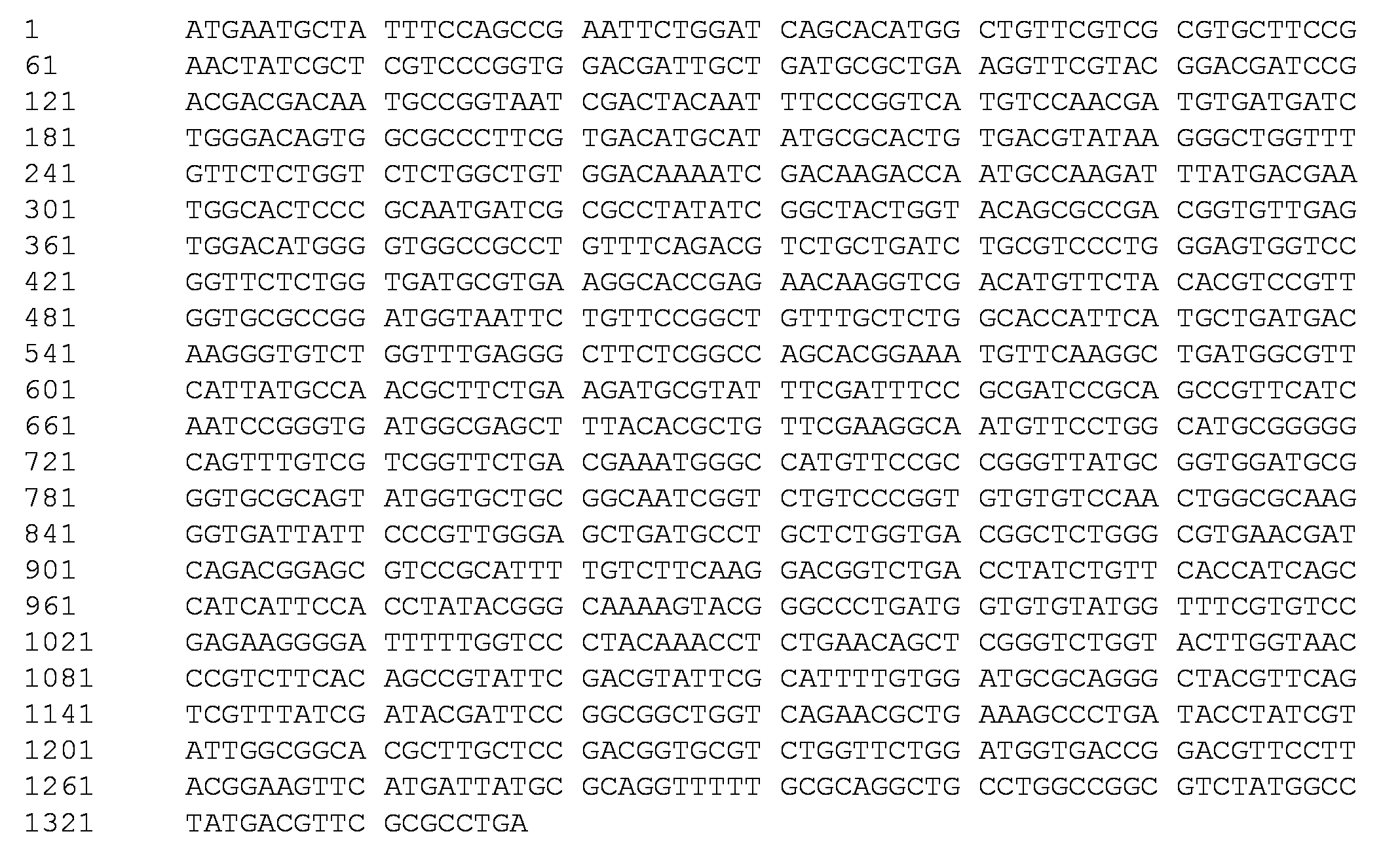


**Fig. S1.** The *levs* sequence of strain MP2116.

Supplement: Multimedia component 2 [file mmc2.docx]
