# Supplementary material for: Enhancing levan biosynthesis by destroying the strongly acidic environment caused by membrane-bound glucose dehydrogenase (mGDH) in Gluconobacter sp. MP2116
Source: Synth Syst Biotechnol. 2024 Aug 20;10(1):68–75. doi: 10.1016/j.synbio.2024.08.005 (PMC11388042; doi:10.1016/j.synbio.2024.08.005)
Supplement: Multimedia component 3 [file mmc3.docx]

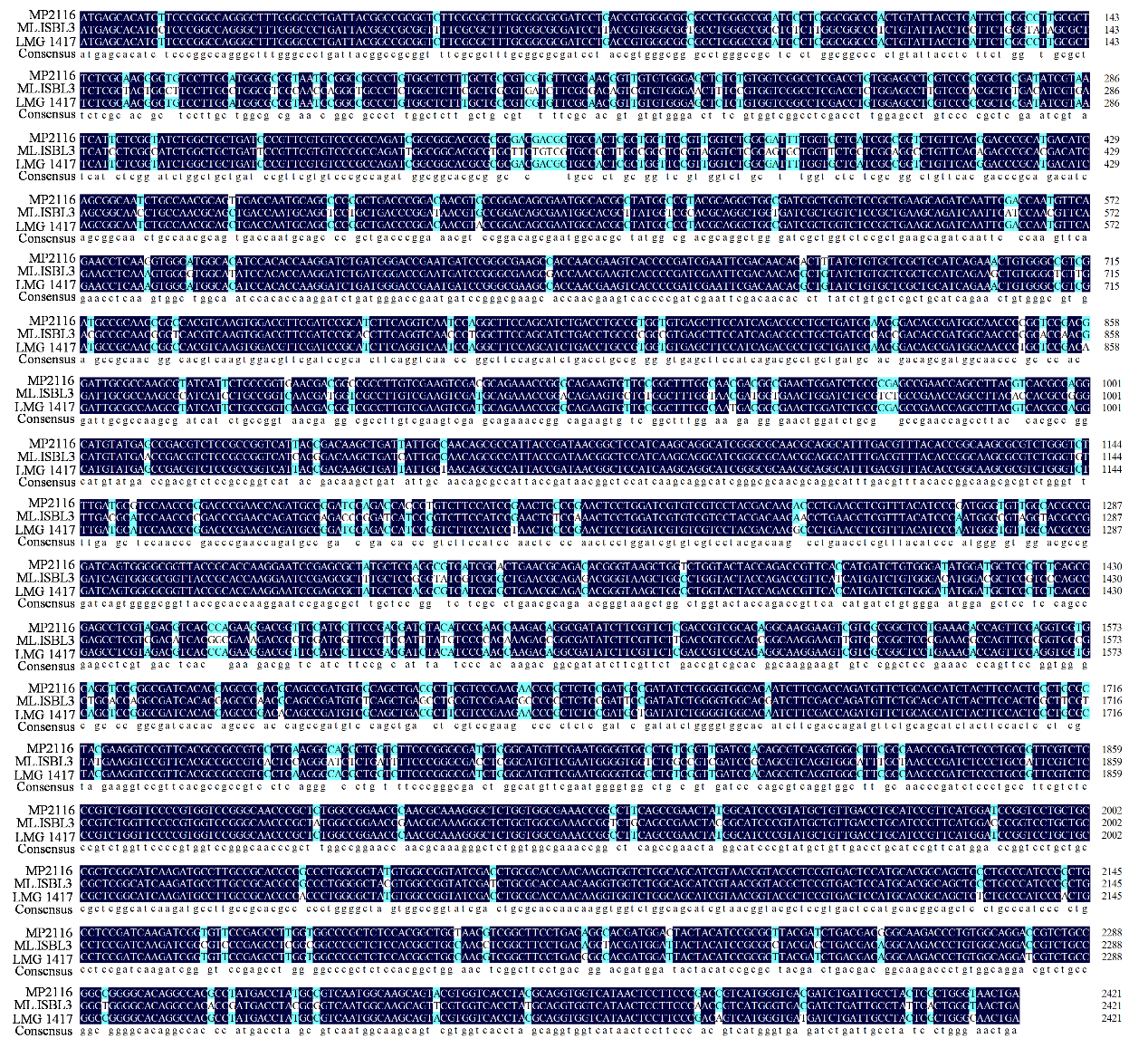


**Fig. S2.** Multiple alignments of the *mgdh* gene from strains MP2116, LMG 1417 (NZ_LHZJ01000078.1/WP_010501939.1) and ML.ISBL3 (CP092689.1/UMM08771.1) using DNAMAN 6.0.
