# Supplementary material for: Enhancing levan biosynthesis by destroying the strongly acidic environment caused by membrane-bound glucose dehydrogenase (mGDH) in Gluconobacter sp. MP2116
Source: Synth Syst Biotechnol. 2024 Aug 20;10(1):68–75. doi: 10.1016/j.synbio.2024.08.005 (PMC11388042; doi:10.1016/j.synbio.2024.08.005)
Supplement: Multimedia component 4 [file mmc4.docx]

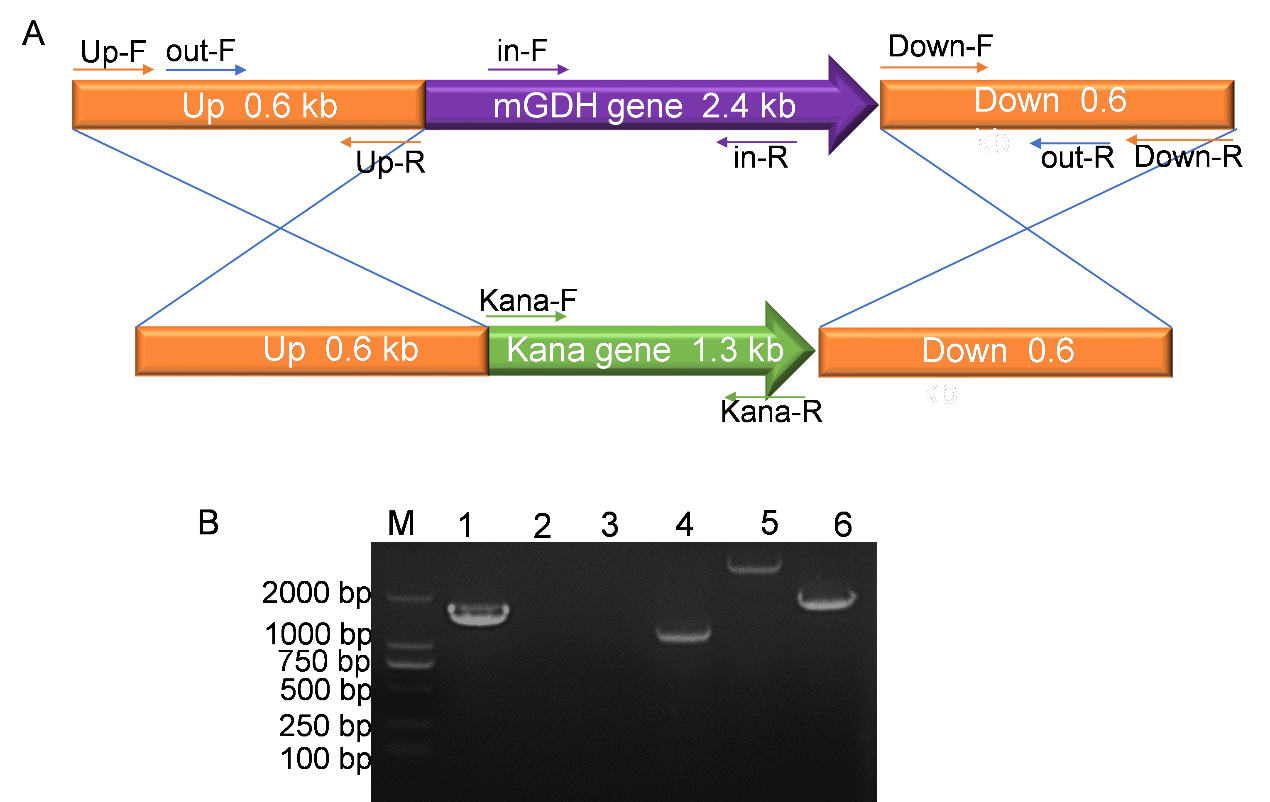


**Fig. S3.** Disruption of the *mgdh* gene in MP2116 and validation of the mutant Δ*mgdh* by PCR. (A) Work procedure used for disruption of the *mgdh* gene in MP2116. (B) PCR products with in-F/R (lanes 1 and 2), Kana-F/R (lanes 3 and 4) and out-F/R (lanes 5 and 6) from the genomic DNAs of MP2116 (lanes 1, 3 and 5) and Δ*mgdh* (lanes 2, 4 and 6).
